# MAVS signaling shapes microglia responses to neurotropic virus infection

**DOI:** 10.1186/s12974-024-03258-6

**Published:** 2024-10-18

**Authors:** Olivia Luise Gern, Andreas Pavlou, Felix Mulenge, Lena Mareike Busker, Luca Ghita, Angela Aringo, Bibiana Costa, Julia Spanier, Inken Waltl, Martin Stangel, Ulrich Kalinke

**Affiliations:** 1 Institute for Experimental Infection Research, Centre for Experimental and Clinical Infection Research, TWINCORE, Joint Venture between the Helmholtz Centre for Infection Research and the Hannover Medical School, 30625 Hannover, Germany; 2 Department of Pathology, University of Veterinary Medicine Hannover, Foundation, 30559 Hannover, Germany; 3 Genentech, South San Francisco, CA USA; 4 Department of Neurology, Hannover Medical School, 30625 Hannover, Germany; 5 Present Address: Translational Medicine Neuroscience, Biomedical Research, Novartis Pharma AG, Basel, 4056 Switzerland; 6 Center of Systems Neuroscience, Hannover, Germany; 7 Cluster of Excellence-Resolving Infection Susceptibility (RESIST, Hannover Medical School, Carl-Neuberg-Straße 1, 30625 Hannover, Germany

**Keywords:** Microglia, MAVS, IPS-1, VISA, Cardif, Virus, CNS

## Abstract

Viral encephalitis is characterized by a series of immunological reactions that can control virus infection in the brain, but dysregulated responses may cause excessive inflammation and brain damage. Microglia are brain-resident myeloid cells that are specialized in surveilling the local CNS environment and in case of viral brain infection they contribute to the control of the infection and to restriction of viral dissemination. Here, we report that after exposure to neurotropic vesicular stomatitis virus (VSV), murine in vitro microglia cultures showed rapid upregulation of a broad range of pro-inflammatory and antiviral genes, which were stably expressed over the entire 8 h infection period. Additionally, a set of immunomodulatory genes was upregulated between 6 and 8 h post infection. In microglia cultures, the induction of several immune response pathways including cytokine responses was dependent on mitochondrial antiviral-signaling protein (MAVS). Consequently, in *Mavs*-deficient microglia the control of virus propagation failed as indicated by augmented virus titers and the accumulation of viral transcripts. Thus, in the analyzed in vitro system, MAVS signaling is critically required to achieve full microglia activation and to mediate profound antiviral effects. In *Mavs*-deficient mice, intranasal VSV instillation caused higher disease severity than in WT mice and virus dissemination was noticed beyond the olfactory bulb. Virus spread to inner regions of the olfactory bulb, i.e., the granular cell layer, correlated with the recruitment of highly inflammatory non-microglia myeloid cells into the olfactory bulb in *Mavs*^*−/−*^ mice. Furthermore, increased cytokine levels were detected in the nasal cavity, the olfactory bulb and in other brain regions. Thus, microglial MAVS signaling is critically needed for virus sensing, full microglia activation, and for orchestration of protective immunity in the virus-infected CNS.

**Supplementary Information:**

The online version contains supplementary material available at 10.1186/s12974-024-03258-6.

## Background

Viral encephalitis is a rare, but serious condition with high mortality and morbidity [1]. A broad range of viruses has neurotropic potential including rabies virus [2], flaviviruses such as tick-borne encephalitis virus (TBEV) [3], West Nile virus (WNV) [4] and Japanese encephalitis virus (JEV) [5], as well as herpesviruses [6] that can gain access to the central nervous system (CNS) and then may cause detrimental disease [7]. The therapeutic arsenal against viral encephalitis, especially caused by RNA viruses, still is very limited and efficient vaccines as well as antiviral reagents are hardly available.

Upon virus entry into the CNS, tissue-resident cells may get infected in a productive or abortive manner and virus sensing through pattern recognition receptors (PRRs) induces the transcription of complex combinations of host genes. During RNA virus infection, the family of cytosolic RNA sensing retinoic acid inducible gene I (RIG-I)-like receptors (RLRs) is of pivotal significance. This protein family consists of RIG-I, melanoma differentiation-associated protein 5 (MDA5), and laboratory of genetics and physiology 2 (LGP2) [8]. RIG-I and MDA5 activate the adaptor molecule mitochondrial antiviral-signaling protein (MAVS) [9–11]. MAVS signals via TANK-binding kinase 1 (TBK1) and IκB kinase-ɛ (IKKɛ) and further downstream by interferon regulatory factor 3 or 7 (IRF3/7), nuclear factor ‘kappa-light-chain-enhancer’ of activated B-cells (NF-κB), and activator protein 1 (AP-1) to induce type I interferon (IFN-I) responses [9]. IFN-I binds to the IFN-I receptor (IFNAR) in an autocrine and/or paracrine manner and IFNAR signals via Janus kinases (JAK) – signal transducer and activator of transcription (STAT) to induce transcription of IFN-stimulated genes (ISGs), which confer antiviral effects [12].

Microglia are the most abundant tissue-resident myeloid cells of the CNS and they are involved in a variety of homeostatic processes such as synapse pruning and remodeling, cellular debris clearance, neurogenesis, vasculogenesis, and resolution of inflammation [13–20]. Within the infected CNS, microglia are of key relevance to restrict virus replication and to limit virus spread as shown by impaired RNA virus control in mouse models with reduced microglia either due to IL-34 deficiency or to pharmacological microglia depletion [21–25]. Moreover, MAVS signaling was shown to have a significant role during West Nile and Rift-Valley fever viral encephalitis highlighting the potential involvement of microglia in the protection against neuroinfection and restriction of viral spread by cytokine responses including IFN-I [26, 27]. However, little is known about the function of MAVS signaling in microglia and whether it is involved in conferring immunopathology of the CNS.

Here, we investigated microglia responses to vesicular stomatitis virus (VSV) infection both in in vitro cultures as well as in vivo, i.e., we studied responses of microglia in their physiological CNS context. Upon VSV exposure of microglia cultures, *Mavs*-deficient microglia showed reduced induction of cytokine responses and de-enrichment of genes that are involved in pro-inflammatory pathways. Consequently, viral loads were higher in *Mavs*^*−/−*^ than in WT microglia cultures as also illustrated by the enhanced abundance of viral transcripts in *Mavs*^*−/−*^ microglia and increased viral titers in cell-free supernatants. Similarly, *Mavs*-deficient mice that were intranasally (i.n.) instilled with VSV showed increased disease severity. Restriction of virus dissemination was impaired and enhanced virus loads in the CNS correlated with increased infiltration with myeloid cells. Despite alternative virus sensing mechanisms being in place, microglia in *Mavs*-deficient mice showed aberrant responses that were not compensated by the highly activated infiltrating myeloid cells pointing towards a key role of MAVS signaling in microglia as a regulator of protective CNS immunity.

## Materials and methods

### Mice

C57BL/6J (wild type or WT) and *Mavs*^*−/−*^ (B6.STOCK-Mavs^(tm1Tsc)^) [28] mice were used in the study. All mice were bred under specific pathogen-free conditions in the central mouse facility of the Helmholtz Centre for Infection Research, Brunswick and at TWINCORE, Centre for Experimental and Clinical Infection Research, Hanover, Germany. Mouse experimental work was carried out using mice older than 8 weeks in compliance with regulations of the German animal welfare law and with the animal experimental protocols that were approved by the Lower Saxony State Office for Consumer Protection and Food Safety (Niedersächsisches Landesamt für Verbraucherschutz und Lebensmittelsicherheit) with protocol numbers 12/1025, 14/1594, 18/2899, and 19/3292.

### Perfusion of mice

For perfusion, mice were deeply anesthetized by intraperitoneal injection of 1.6% ketamine and 0.08% xylazin in physiological saline (0.3334 mg/g ketamine and 0.01668 mg/g xylazin, 200 µL/10 g mouse body weight). Mice were fixated horizontally to a block and transcardially perfused with 10 mL of ice-cold PBS when breathing had stopped. For preparation of brains for histological analysis, mice were first perfused with 10 mL of PBS and then with 10 mL of 4% PFA.

### Mixed glial culture and microglia isolation

The isolation of primary murine microglia was performed as previously described [29]. In brief, newborn mice were decapitated between day 2–4 post-natum (hereafter referred to as “P3 pups”), cortices were collected and dissected by removing blood vessels and meninges with forceps under a stereomicroscope (Nikon, SMZ45T). The tissue was minced and digested in 0.1% trypsin (Sigma-Aldrich) and 0.25% DNAse (Roche). The resulting single-cell suspension was seeded in poly-L-lysine (Sigma-Aldrich) precoated flasks in DMEM (Capricorn) with 1% penicillin/streptomycin (Gibco) supplemented with 10% fetal calf serum (FCS, Capricorn) and cultivated at 37 °C in 5% CO_2_. Medium was changed on day 1 and 5. Microglia were collected after 9–11 days by shaking the flasks at 37 °C at 135 rpm on an orbital shaker for 40 min. The medium was collected and microglia were seeded at a density of 2 × 10^5^ cells per well into a 24-well plate or on glass coverslips, or at a density of 5 × 10^4^ into a 96-well plate and used the following day for experiments.

### Virus

VSV-Indiana (Mudd-Summers isolate) was originally obtained from D. Kolakofsky (University of Geneva, Switzerland). VSV-eGFP was obtained by G. Zimmer (University of Bern, Switzerland) [30].

### VSV infection of mice

Mice were anaesthetized by intraperitoneal injection of 1.6% ketamine and 0.08% xylazin in physiological saline (0.1667 mg/g ketamine and 0.00834 mg/g xylazin, 100 µL/10 g mouse body weight). Mice were intranasally (i.n.) instilled with a total volume of 10 µL containing 1000 plaque forming units (PFU) of VSV Indiana (Mudd Summers isolate) or VSV-eGFP in sterile PBS in both nostrils. Treated mice were monitored and scored daily in a non-blinded manner. A score of 1 represents healthy animals without signs of disease. A score of 2 represents animals with slightly reduced activity and slightly reduced exploratory behavior. A score of 3 represents sick animals with shaggy fur and hunchback posture. At a score of 3, animals were taken out of the experiment. A score of 4 would have indicated severely sick animals showing signs of hind limb paralysis or flattened breathing. A score of 4 was not reached in any animal of the performed experiments.

### VSV infection of microglia cultures

Microglia were in vitro exposed to VSV-Indiana at an MOI of 0.5 in pure DMEM medium or they were mock-treated by medium exchange with pure DMEM and the cells were incubated for 1 h at 37 °C in 5% CO_2_. 1 h post treatment, medium was removed from infected and mock-treated wells, followed by washing with PBS and addition of fresh medium containing 10% FCS and 1% penicillin/streptomycin. Addition of fresh medium marks the 0 h time point.

### RNA isolation and bulk RNA-seq

RNA was isolated from WT and *Mavs*^*−/−*^ microglia that were either mock-treated or VSV-infected for 2, 4, 6, or 8 h using Trizol. Total RNA was extracted using Direct-zol RNA Miniprep Plus Kit (Zymo Research) according to the manufacturer’s instructions. RNA quality and integrity were assessed with Bioanalyzer 2100 (Agilent) and samples with RIN value > 8.0 were selected for further processing. Sequencing libraries were constructed and processed on Illumina NovaSeq-600 platform with 50 bp paired-end read configuration. Quality control of the sequenced raw FASTQ files was performed with the FastQC software (version 0.11.9) and the data were mapped to the Ensembl mouse genome reference version GRCm38 (mm10) or VSV Indiana reference genome using STAR [31] (version 2.5.4b). Gene quantification was determined also by STAR and genes with a maximum read count < 10 were removed from all samples before further analysis. Differential expression analysis was performed with the DESeq2 package [32] and in the R environment (version 4.3.1), setting the FDR to 0.05 with test p-values adjusted using the Benjamini-Hochberg procedure. To display expression levels of selected gene signatures between samples, raw counts were normalized based on median of ratios method in DESeq2. In brief, DESeq2 generates normalized reads by dividing counts with sample-specific size factors that were determined by the median ratio of gene counts relative to the geometric mean per gene. Functional annotation and enrichment analysis was performed [33] on the sets of differentially regulated genes using the Gene Ontology (GO) and Reactome databases using ClusterProfiler Bioconductor package [34].

### Immunocytochemistry and microscopy

Coverslips were fixed in 4% paraformaldehyde (PFA) for 20 min at room temperature (RT), washed, and stored in PBS. Non-specific antibody binding was prevented by incubation with blocking buffer containing 3% donkey serum (Sigma-Aldrich) and 0.3% Triton (Sigma-Aldrich) in PBS for 1 h at RT. Then, the primary antibodies in blocking buffer were added overnight at 4 °C. Unbound antibodies were removed by 3 washing steps with PBS before incubation with the secondary antibodies in blocking buffer for 1 h at RT. Nuclei were counterstained with DAPI (4′,6-diamino-2-phenylindole, 1:10,000, Thermo Fisher Scientific, RRID: AB_2307445) and the coverslips were mounted with mounting medium (DAKO) on glass slides. Images were taken on Olympus FV1000 or Olympus FV3000 confocal microscopes with the FV31S-SW software and analyzed by Fiji. Primary antibodies: rabbit anti-mouse Iba1 (Wako, 1:200), mouse anti-mouse GFAP (Millipore, 1:400). Secondary antibodies: donkey anti-rabbit AF568 (Invitrogen, 1:500), donkey anti-mouse AF647 (Invitrogen, 1:500).

### Spectral flow cytometry

In vitro cultured microglia were harvested, washed in PBS, and stained with Zombie-Aqua live/dead fixable dye (BioLegend). Unspecific immunolabeling conferred by Fc-receptor binding was blocked by the addition of anti-CD16/CD32 (BD Pharmingen). For the characterization of the surface phenotype of microglia, cells were immunolabeled with BUV395-conjugated anti-mouse CD45 (1:50, clone 30-F11, BD Horizon), BV421 anti-mouse F4/80 (1:25, clone BM8, Biolegend), BV605-conjugated anti-mouse CSF-1R (1:50, clone AFS98, Biolegend), PE-conjugated anti-mouse CX3CR1 (1:25, clone SA011F11, Biolegend), PE-Cy7-conjugated anti-mouse CD11b (1:25, clone M1/70, Biolegend), and APC-conjugated anti-mouse P2RY12 (1:25, clone S16007D, Biolegend) antibodies for 20 min at 4 °C in the dark. APC-conjugated anti-mouse P2RY12 antibody was exchanged with APC-conjugated anti-mouse GLAST (1:25, clone ACSA-1, Miltenyi) or with APC-conjugated anti-mouse O4 (1:50, clone # 04, R&D). Data were acquired using an ID7000 Spectral Cell Analyzer (Sony) and analysis was performed using the FlowJo version 10 software (Tree Star Inc.).

### Preparation of whole head sections for microscopy

After perfusion of mice, the entire head was collected. Skin and jaws were removed and the head was incubated overnight in 4% PFA in a 15 mL Falcon tube. The head was placed in decalcification buffer (0.1 M phosphate buffer, 10 g sucrose, 160 g EDTA; pH ≥ 7,4 for 1 L) for 5–7 days at 50 °C under constant shaking at 50 rpm. The skull was carefully opened and the head was processed through 10%, 20%, and 30% sucrose solutions for 1 day at 4 °C, each. The head was then frozen in OCT (Tissue-Tek) and stored at -20 °C until slicing. 16–20 μm slices were cut with the cryotome (NX 70, Thermo Fisher Scientific). Brain slices were counterstained with DAPI (1:1000) and coated with mounting medium (DAKO). Images were taken on an Axio scan.Z1 microscope (Zeiss). Adjustment for brightness, contrast and color balance was done using ZEN and Fiji software.

### Determination of virus titers by plaque assay

For quantification of infectious virus particles in brain regions, mice were perfused with PBS, brains were taken and separated into olfactory bulb (OB), cerebrum (CR), cerebellum (CRBL), and brain stem (BS). Additionally, the nasal cavity (NC), spinal cord (SC), lung, and liver were collected. The organs were collected in Lysing Matrix tubes (MP Biomedicals) containing MEM medium (5% FCS) and stored at − 80 °C. After thawing and homogenization in a sample homogenizer (MP Biomedicals), organ homogenates were transferred on monolayers of Vero cells in serial 10-fold dilutions. Following 1 h incubation at 37 °C, cells were overlaid with MEM containing 1% methylcellulose. After incubation for 24 h at 37 °C, the overlay was removed, the monolayer was fixed and stained with 0.5% crystal violet and plaques were counted.

### Immunohistochemistry and microscopy of organ slices

Brains were collected from animals, fixed with 4% PFA for 2 h at 4°C, and incubated overnight in 30% sucrose. Left and right brain hemispheres were separated, embedded in OCT, and then stored at − 20°C. 7 µm thin sagittal sections were cut using a cryotome. For immunolabelling, the tissues were rehydrated with 0.5% Triton X-100 in PBS for 15 min. Blocking was performed with 5% donkey serum and 0.5% Triton X 100 in PBS for 1 h at RT. The tissues were incubated with primary antibodies overnight at 4°C. After washing with 0.5% Triton X-100 in PBS, the tissues were incubated with secondary antibodies for 1 h at RT in the dark, incubated with DAPI (1:1000) for 2 min at RT in the dark, washed with 0.5% Triton X-100 in PBS, and ultimately mounted with mounting medium (DAKO). Primary antibodies: goat anti-mouse Iba1 (Abcam, 1:500), rabbit anti-mouse P2RY12 (AnaSpec, 1:500), rat anti-mouse Mac-3/CD107b (BD Biosciences, 1:250), rat anti-mouse/human Mac-2/Galectin-3 (Cedarlane, 1:500). Secondary antibodies: donkey anti-goat AF488 (Invitrogen, 1:1000), donkey anti-rat AF 568 (Abcam, 1:1000), donkey anti-rabbit AF647 (Invitrogen, 1:1000). Images were taken with a confocal microscope (Olympus FV3000, software FV31S-SW). Brightness was adjusted, background noise reduced, and total numbers of Iba1^+^ cells, Iba1^+^ P2RY12^+^ cells, Iba1^+^ P2RY12^−^ cells as well as Iba1^+^ Mac-3^+^, Iba1^+^ P2RY12^+^ Mac-3^+^, and Iba1^+^ P2RY12^−^ Mac-3^+^ cells per mm^2^ of the glomerular layer of the olfactory bulb were quantified by Fiji software.

### Cytokine array

The cytokine content of organ homogenates of the NC, OB, BS, and CRBL was determined by using the LEGENDplex™ mouse inflammation panel 1 kit (BioLegend), following the manufacturer’s instructions. Data were acquired using a ID7000 Spectral Cell Analyzer (Sony) and analysis was performed on LEGENDplex™ Version 2023-02-15 software (BioLegend).

### Quantitative RT-PCR

For real-time quantitative analysis, RNA was extracted from OB using the NucleoSpin RNA Kit (Macherey-Nagel) and complementary DNA (cDNA) synthesized by using PrimeScript RT Master Mix (TaKaRa) according to the manufacturer’s instructions. The expression levels of *P2ry12*, *Fcgr1*, and *Gapdh* were quantified with SYBR Green (Bioline) in a LightCycler 480 (Roche) using the following primers: *P2ry12* (CATTGACCGCTACCTGAAGACC; GCCTCCTGTTGGTGAGAATCATG), *Fcgr1* (AGAAGCGATGGCGTGTATGA; TTCAGGCTGCTCGGGTCCAC), and *Gapdh* (TGCACCACCAACTGCTTAGC; GGCATGGACTGTGGTCATGAG). Expression levels of *P2ry12* and *Fcgr1* were normalized against the housekeeping gene *Gapdh* and fold-change (FC) was assessed using the 2^−ΔΔCt^ method.

### Statistical analysis

Statistical tests were performed using GraphPad Prism 10 Vers. 10.1.1 (270, GraphPad Software Inc) as indicated in the respective figure legends. Reproducibility of experiments was determined in independent experiments as indicated in the figure legends. The experiments were not randomized. The investigators were not blinded to allocation during the experiments and the outcome assessment. P values of 0.05 and below were considered statistically significant.

## Results

### Primary murine microglia cultures are a versatile model to study antiviral innate responses

**Fig. 1:**
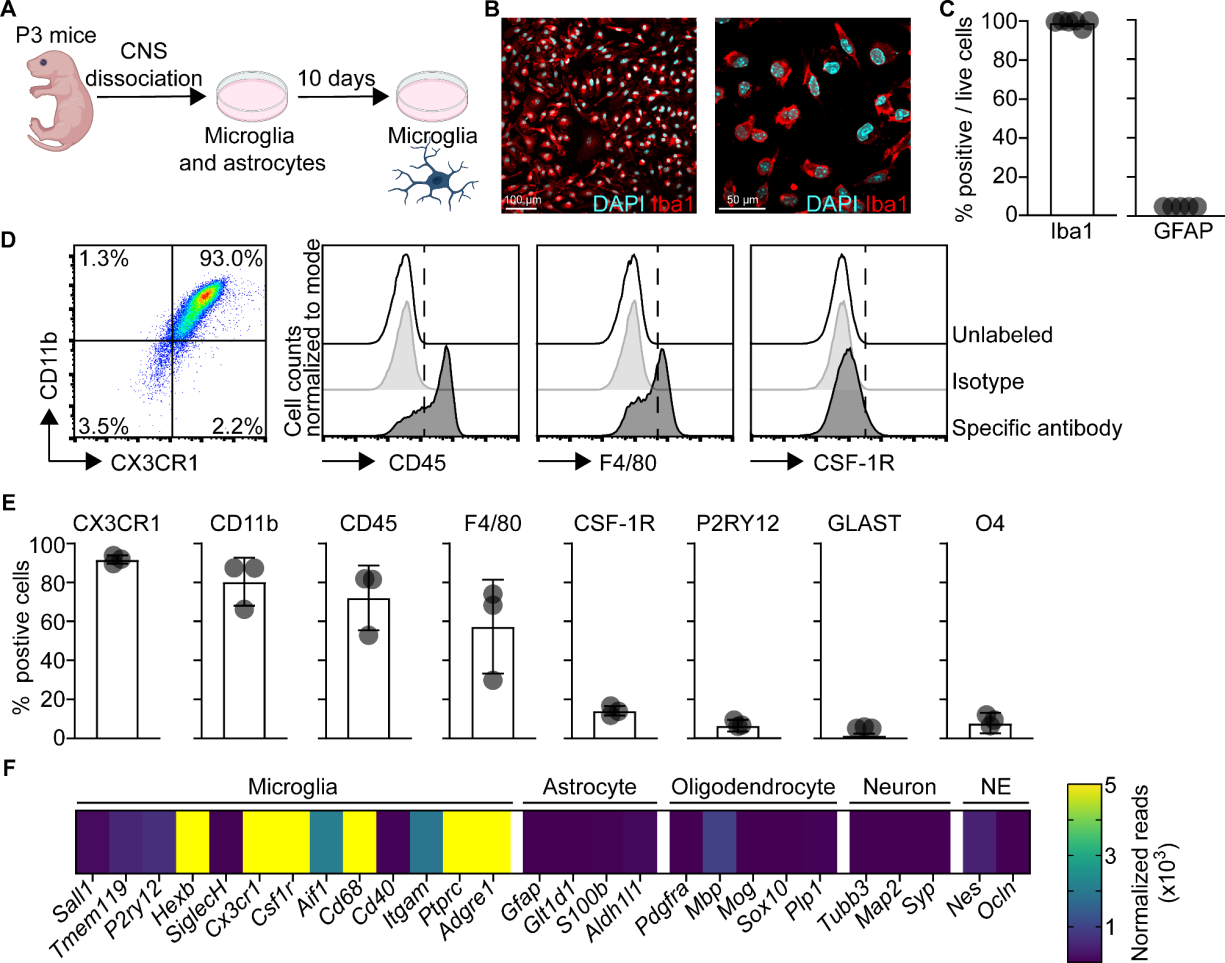
Primary murine microglial cultures express several microglia core genes and display a high degree of purity. **A** Schematic depiction of the generation of murine mixed glial cultures and isolation of microglia. **B** Immunofluorescent confocal microscopy of isolated microglia immunolabelled with Iba1 (red) and counterstained with DAPI (cyan). Objective 20x (left) or 60x (right). **C** Quantification of Iba1^+^ cells amongst live cells and GFAP^+^ cells (*N* = 3 independent microglia preparations, *n* = 5–6 photographs quantified). **D** Spectral flow cytometric analysis of immunolabeled in vitro microglia. Data of live cells of one representative preparation is shown. **E** Quantification of positive cells for the depicted surface markers characteristic for microglia (CX3CR1, CD11b, CD45, F4/80, CSF-1R, and P2RY12) or astrocytes (GLAST) or oligodendrocyte progenitors (O4) (*n* = 3 independent microglia preparations). **F** Bulk RNA-seq analysis of microglia cultures reveals expression of several microglia-specific genes and absence of gene expression characteristic for other CNS resident cell types (astrocytes, oligodendrocytes, and neurons). Isolated microglia were plated, harvested, and total RNA was isolated to perform RNA-seq analysis. Normalized transcript counts are depicted as mean of technical triplicates (*n* = 3 technical replicates)

To study the influence of neurotropic virus infection on microglia, we retrieved microglia from murine primary glial cell cultures. In brief, we isolated brain cortices from P3 mouse pups, cultivated the mixed glial cells in vitro for 10 days, and then harvested microglia (Fig. 1A). The purity and identity of microglia cultures were further assessed by immunofluorescence microscopy, flow cytometry, and RNA sequencing (RNA-seq). Immunolabeling and fluorescence microscopy of cultured microglia revealed that basically all live cells were Iba1^+^ and GFAP^−^ and showed the characteristic morphology of myeloid cells (Fig. 1B-C, Fig. S1A). Astrocytes were used as a positive control for the detection of GFAP expression (Fig. S1B). Flow cytometric analysis with stringent hierarchical gating of cells, singlets, and live cells (Fig. S1C) revealed abundant expression of CX3CR1, CD11b, and CD45 and approximately 50% of cultured microglia expressed F4/80 (Fig. 1D-E). In contrast, low and undetectable expression levels of CSF-1R and P2RY12 were found, respectively (Fig. 1D-E). Moreover, the astrocytic marker GLAST as well as the oligodendrocyte precursor marker O4 were absent (Fig. 1E). To substantiate the flow cytometric data, we performed transcriptomic analysis of mock-treated microglia cultures. Quantification of normalized reads revealed high expression of several myeloid marker genes including *Cd68* and *Itgam* (CD11b) as well as the classical myeloid cell markers *Cx3cr1*, *Aif1* (Iba1), *Adgre1* (F4/80), and *Csf1r* (Fig. 1F). Microglia markers that are abundantly expressed in adult mice such as *Sall1*, *Tmem119*, *SiglecH*, and *P2ry12* were detected at relatively low levels, which is in accordance with other studies using microglia cultures [35]. The purity of microglia cultures was further underscored by absence of the astrocytic marker genes *Gfap*, *S100b*, and *Aldh1l1*, the oligodendrocytic marker genes *Pdgfra*, *Mbp*, and *Plp1*, and the neuronal marker genes *Tubb3*, *Map2*, and *Syp* (Fig. 1F). Furthermore, genes characterizing cells emerging from the neuroectodermal (NE) lineage such as *Nes* and *Ocln* were absent as well. Collectively, our data demonstrated that the microglia cultures used in this study mainly consist of microglia and that they are a robust and amendable model to investigate antiviral responses of microglia.

### After in vitro exposure to virus, microglia show a pronounced transcriptional shift towards inflammation and the induction of immunomodulatory genes

**Fig. 2:**
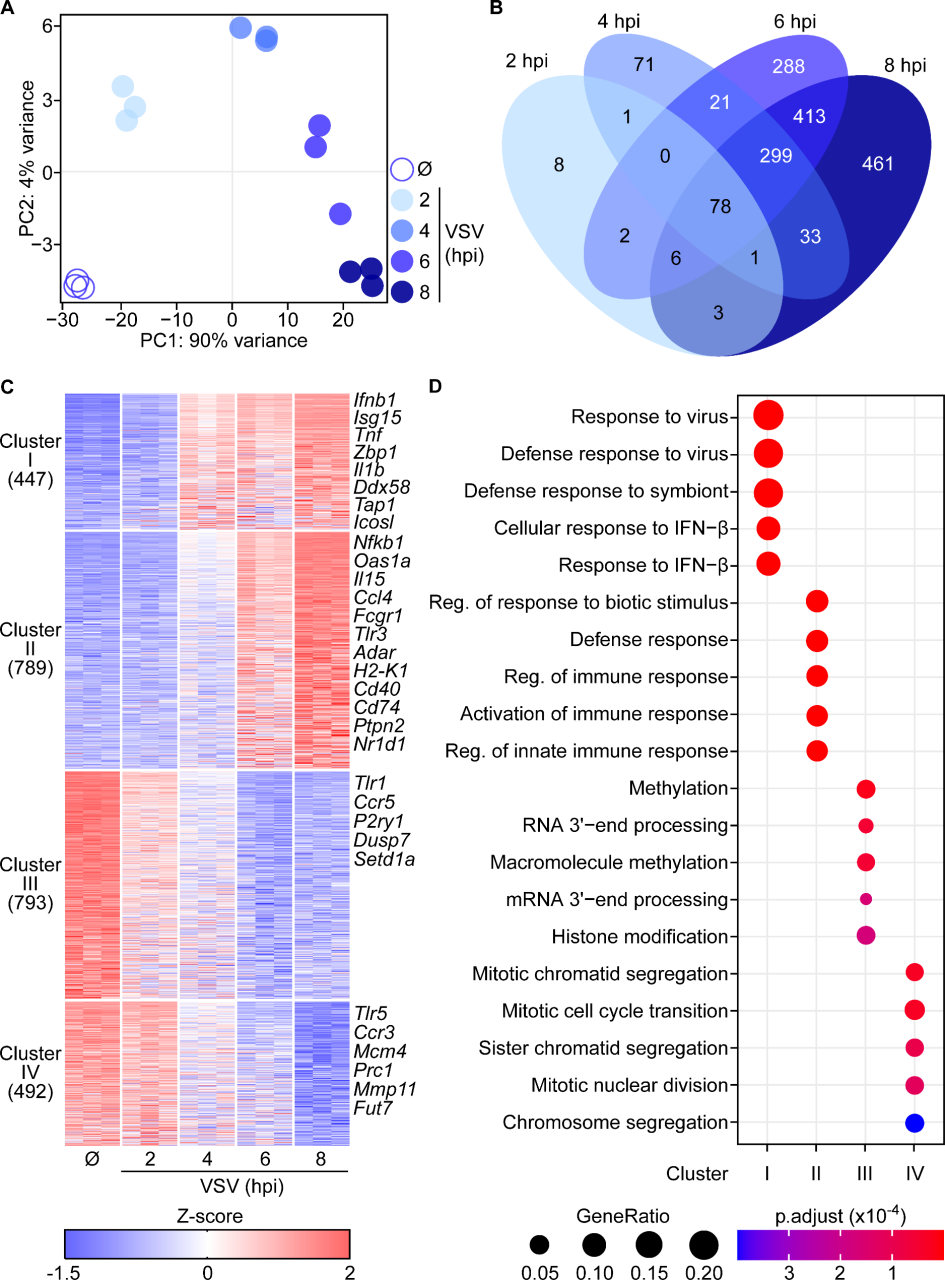
Cultured microglia respond rapidly to VSV exposure by upregulation of pro-inflammatory genes. WT in vitro microglia were isolated, plated, and either infected with VSV at an MOI of 0.5 or mock-treated (Ø). At indicated time points, microglia were harvested, and total RNA was isolated for bulk RNA-seq analysis. **A** Principal component analysis (PCA) of mock-treated controls (Ø) and VSV-infected microglia at indicated time points. **B** Venn diagram of differentially expressed genes (DEGs, log_2_ fold-change > │2│, p_adj_ < 0.05) between either VSV-infected at ‘2 hpi’, ‘4 hpi’, ‘6 hpi’ or ‘8 hpi’ vs. the mock-treated control. **C** Heatmap of k-means clustering of DEGs (log_2_ fold-change > │2│, padj < 0.05). Each column represents transcripts from a technical replicate. **D** Pathway analysis of 4 clusters visualized in the heatmap. Cluster I and II comprise genes that are upregulated after VSV infection; cluster III and IV comprise genes that are downregulated after VSV infection. *n* = 3 technical replicates

To better understand microglial responses to viral infection, microglia cultures were mock-treated (Fig. 1F) or they were exposed to VSV Indiana at MOI of 0.5 for 2, 4, 6, and 8 h and RNA-seq was performed. Principal component analysis (PCA) showed a clear clustering of samples from the different experimental groups, indicating substantial transcriptional changes of microglia during the course of virus infection (Fig. 2A). Among the VSV-infected samples, the 2 and 4 h samples largely segregated from each other and the other samples, whereas the separation was less dominant for the 6 and 8 h samples. This time course analysis revealed transcriptional dynamics that corresponded with early and late microglia anti-viral responses. To further investigate these changes, we performed pairwise comparison between VSV-exposed samples and the mock controls. By applying the selection criterion of log2 fold change > |2|, padj < 0.05, we identified 99, 504, 1107, and 1294 genes that were differentially expressed after 2, 4, 6, and 8 h of VSV exposure, respectively (Fig. 2B). Of these genes, 78 were commonly shared across all the time points, whereas 8, 71, 288, and 461 genes were uniquely expressed after 2, 4, 6, and 8 h of infection, respectively.

Using unbiased k-means clustering of differentially regulated genes, we identified four distinct clusters (Fig. 2C). Cluster I comprised many antiviral mediators (*Ifnb*, *Isg15*, *Tnf*, *Zbp1*, and *Il1b*), the RNA sensor *Ddx58* (RIG-I) as well as genes that are involved in antigen presentation (*Tap1* and *Icosl*), which were enriched from 4 hpi onwards and therefore can be annotated as early antiviral response genes. Cluster II comprised genes encoding for central antiviral factor *Nfkb1*, ISGs such as *Oas1a*, cytokines and chemokines (*Il15* and *Ccl4*), phagocytosis receptor *Fcgr1*, innate immune sensors (*Tlr3*), factors involved in discriminating self and non-self RNA (*Adar*), antigen presentation and co-stimulation (*H2-K1*, *Cd40*, and *Cd74*) as well as immunoregulatory molecules (*Ptpn2* and *Nr1d1*) (Fig. 2C). Since enrichment of genes in cluster II started at 6 hpi and was further enhanced at 8 hpi, cluster II can be annotated as late immune response genes. Cluster III and IV comprised genes that were de-enriched after VSV infection. Cluster III consisted of genes that are involved in the immune response (*Tlr1* and *Ccr5*), the extracellular ATP/ADP sensor *P2ry1*, and factors driving cell division (*Dusp7* and *Setd1a*), which were de-enriched from 6 hpi on. Cluster IV additionally comprised genes that are associated with immune activation (*Tlr5* and *Ccr3*), cell cycle (*Mcm4* and *Prc1*), remodeling of extracellular matrix (*Mmp11*), and metabolism (*Fut7*), which are genes that were de-enriched at the later time point of 8 hpi.

To substantiate the functional phenotype associated with the identified cluster-specific genes, we performed pathway analysis (p-value cut-off < 0.05) using gene ontology (GO) [34]. Cluster I exclusively expressed genes associated with defense responses against virus infection and cellular responses to IFN-β, while cluster II comprised genes involved in activation and regulation of immune responses (Fig. 2D). Notably, cluster III and IV contained genes that are implicated in methylation, RNA processing, nuclear division as well as chromosome segregation. The early response mounted around 4 hpi (cluster I) mostly focused on transcriptional activation of innate immune activating pathways, including IFN responses and preparation for antigen presentation. While most induced genes from cluster I were sustained until 8 hpi, activating as well as immunomodulatory pathways were upregulated with moderately delayed kinetics, i.e., at 6 hpi (cluster II), pointing towards regulation of the cellular response. The early downregulated gene sets (cluster III) include histone modifications, which might lead to regulation of the transcription. While immune activation dominated the transcriptional landscape of VSV-infected microglia, cell division genes ceased at the later time points, i.e., at 6 and 8 hpi (cluster IV). In conclusion, in vitro VSV-exposed microglia mount an immediate innate response characterized by a time-dependent expression of activating and immunomodulatory components as well as the reduction of their cell cycle activities.

### During in vitro VSV infection of microglia, MAVS signaling is critically needed for mounting immune responses

**Fig. 3:**
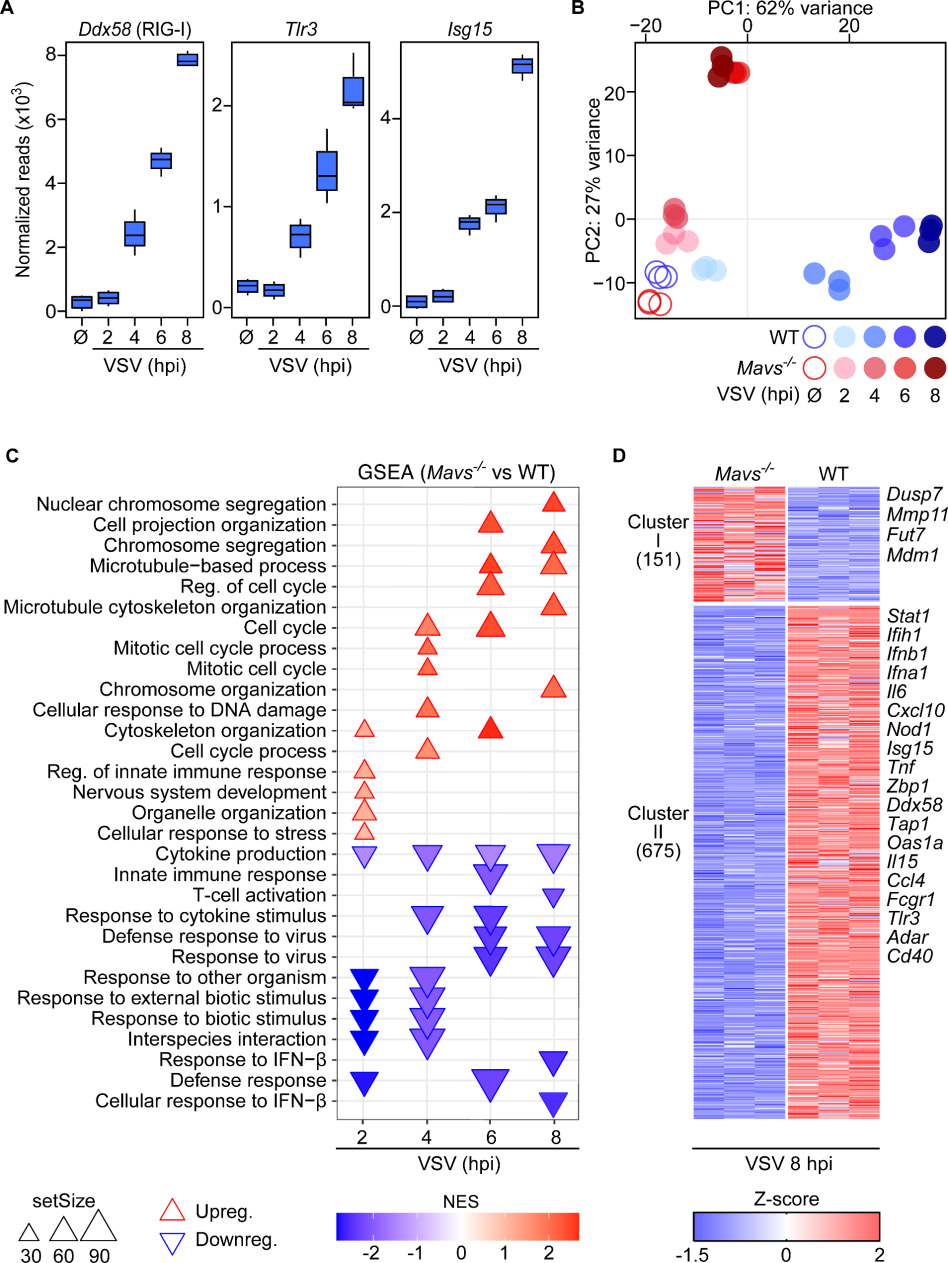
Upon VSV exposure, MAVS is crucial for efficient activation of in vitro microglia. Microglia were isolated, plated, and were either infected with VSV at an MOI of 0.5 or mock-treated (Ø). Cells were harvested and total RNA was isolated to perform bulk RNA-seq analysis. **A** Normalized gene counts of *Ddx58* (RIG-I), *Tlr3*, and *Isg15* of VSV-infected or mock-treated (Ø) WT microglia. Each group contains triplicates. Each box represents interquartile range while whiskers indicate maximum and minimum values. **B** PCA of VSV-infected and mock-treated (Ø) WT and *Mavs*^*−/−*^ microglia at indicated time points. **C** Biological processes (BP) pathway analysis of *Mavs*^*−/−*^ vs. WT microglia exposed to VSV for 2 h, 4 h, 6 h, or 8 h in vitro reveals downregulation of pathways related to immune responses in *Mavs*^*−/−*^ microglia. Red, upright triangles (△) represent upregulated pathways in *Mavs*^*−/−*^ microglia while blue, down-pointing triangles (▽) represent downregulated pathways in *Mavs*^*−/−*^ microglia in comparison to WT microglia. Triangle size represents the gene set size of the annotated pathways. **D** Heatmap of k-means clustering of DEGs (log_2_ fold-change > │2│, padj < 0.05) of *Mavs*^*−/−*^ vs. WT microglia at 8 h post VSV infection in vitro. Each column represents transcripts from a technical replicate. *n* = 3 technical replicates

The identification of GO terms that correspond to defense responses to virus infection in cluster I (Fig. 2D) prompted us to address whether VSV exposure would trigger the microglia sensome. To this end, we sorted and filtered genes based on the aforementioned GO term. This approach resulted in the identification of the ISGs *Ddx58* (RIG-I), *Tlr3*, and *Isg15* that were strongly increased in microglia after VSV exposure (Fig. 3A). Indeed, RIG-I is an important RNA sensor in the cytosol that confers signals via the adapter molecule MAVS to induce IFN responses in infected cells [36]. To assess the functional relevance of the RIG-I signaling axis on microglia during viral infection, we prepared microglia cultures from brain cortices of *Mavs*-deficient P3 mouse pups (hereafter denoted as “*Mavs*^*−/−*^ microglia”) and performed RNA-seq analysis. We first addressed transcriptional dynamics of *Mavs*^*−/−*^ microglia in response to VSV infection. In contrast to WT, cluster-specific genes corresponding to early responses were induced from 6 hpi onwards, while genes involved in chromosome segregation and innate immune responses (*Tbk1*, *Ccl4*, *Il16*, and *Irak2*) were abrogated at 8 hpi (Fig. S2A-B), indicating the induction of dysregulated responses in *Mavs*^*−/−*^ microglia. To further explore transcriptional similarities and differences in WT and *Mavs*^*−/−*^ microglia, we integrated WT and *Mavs*^*−/−*^ microglia samples and performed a combinatorial analysis of the retrieved data. Following quality control, PCA revealed divergent expression profiles of WT and *Mavs*^*−/−*^ cells during the course of VSV infection (Fig. 3B). Infected WT microglia exhibited a stronger shift in gene expression, as highlighted by PC1 accounting for 62% variance, whereas PC2 that corresponded to *Mavs*-deficient microglia showed 27% variance. Closer examination of the clustering of single samples showed low variation between the biological replicates, which indicated that comparison of gene signatures at different time points was possible. Interestingly, samples from mock-treated cells clustered closely together, irrespective of their genotype, suggesting that *Mavs*-deficiency did not significantly affect the homeostatic signature of microglia cultures (Fig. 3B). Both WT and *Mavs*^*−/−*^ microglia exposed to VSV for 2 h clustered in close proximity to their respective mock-treated controls. Notably, there was a major transcriptional change in WT microglia at 4 hpi when compared with the respective mock-treated control. However, this phenotype was less pronounced in *Mavs*^*−/−*^ microglia at 4 hpi indicating a delayed response. The 6 and 8 hpi samples of WT microglia clustered together along PC1, as similarly detected for *Mavs*^*−/−*^ microglia at 6 and 8 hpi, which clustered along PC2, indicating that the major transcriptional modifications have occurred by 6 h after VSV exposure. Differential gene expression analysis revealed increased numbers of differentially expressed genes (DEGs) during the VSV infection time course and confirmed the weaker induction of gene expression of *Mavs*^*−/−*^ microglia at 4 hpi (Fig. S2C). Notably, *Mavs*^*−/−*^ microglia showed a stronger downregulation of genes at 8 hpi than WT microglia. Taken together, these data revealed that VSV-exposed *Mavs*^*−/−*^ microglia mounted weaker transcriptional responses and delayed transcriptional shifts than VSV-exposed WT controls.

To investigate MAVS-dependent effects on microglia responses after virus exposure, we next performed differential gene expression analysis of VSV-infected microglia at 2, 4, 6, and 8 h of *Mavs*^*−/−*^ versus WT microglia and categorized the functional relevance of identified genes by using gene set enrichment analysis (GSEA). This analysis revealed significant de-enrichment of GO terms implicated in innate and adaptive immune responses. Notably, at 2 hpi pathways corresponding to cytokine production, response to external stimuli, and defense response were strongly downregulated in *Mavs*^*−/−*^ microglia (Fig. 3C). At 4 and 6 hpi, *Mavs*^*−/−*^ microglia exhibited de-enrichment of GO terms involved in cytokine stimuli, innate immune response, and response to virus. Remarkably, GO terms associated with cytokine production, T-cell activation, and cellular response to IFN-β were downregulated in *Mavs*^*−/−*^ microglia at 8 hpi (Fig. 3C), further underscoring the importance of MAVS in shaping both innate and adaptive immune responses of the inflamed CNS. The delayed induction and strong downregulation of GO terms associated with cellular response to IFN-β and T-cell activation in *Mavs*-deficient microglia prompted us to examine the nature of genes regulated at 8 h. To address this, we performed pairwise comparison of *Mavs*^*−/−*^ versus WT microglia. By applying the same criterion of significance as above, a total of 826 DEGs were detected, which separated into 2 clusters. Cluster I corresponded to 151 genes that were enriched in *Mavs*^*−/−*^ microglia, including genes related to cell cycle such as *Dusp7* and *Mdm1*, genes involved in remodeling of the extracellular matrix (*Mmp11*), and genes involved in metabolic processes such as *Fut7* (Fig. 3D). Cluster II comprised 675 genes that were de-enriched in *Mavs*^*−/−*^ microglia and that encoding for IFNs (*Ifna1* and *Ifnb1*), IFN signaling (*Stat1*), antiviral mediators (*Isg15*, *Oas1a*, and *Zpb1*), chemokines and cytokines (C*xcl10*,* Ccl4*, *Il6*, *Tnf*, and *Il15*), PRRs (*Nod1*, *Tlr3*, *Ddx58*, *Ifih1* [MDA5]) as well as antigen processing and presentation complexes (*Fcgr1*, *Tap1*, and *Cd40*).

### MAVS is crucial to restrict viral replication in microglia cultures

**Fig. 4:**
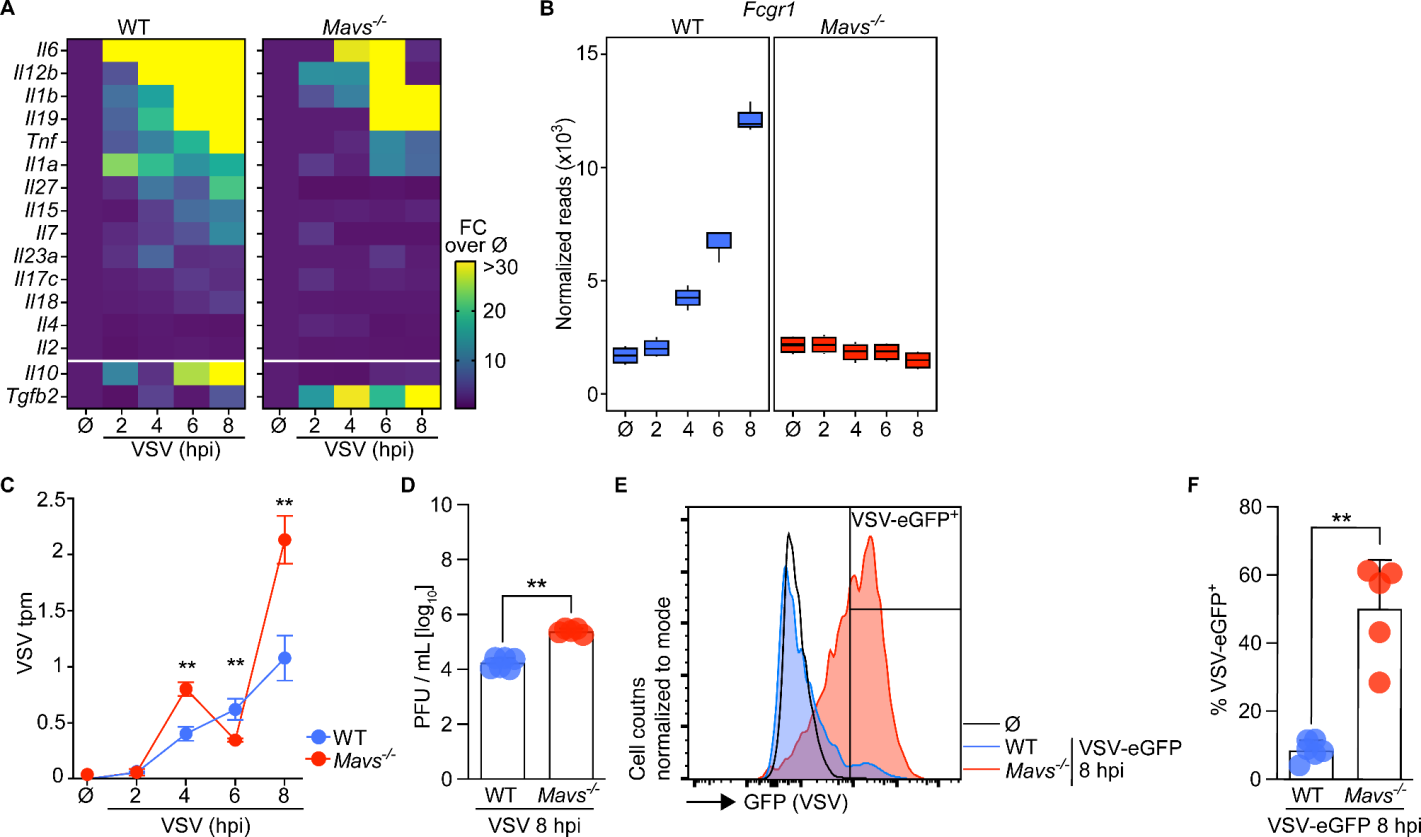
MAVS is needed to restrict VSV infection in microglia cultures. **A** Fold change (FC) of pro-inflammatory cytokine normalized gene counts upon VSV infection in WT or *Mavs*^*−/−*^ microglia compared to mock-treated controls (Ø). Mean values of 3 technical replicates are depicted. **B** Normalized gene counts of *Fcgr1* in VSV-infected and mock-treated (Ø) WT and *Mavs*-deficient microglia. Each group contains technical triplicates. Each box represents interquartile range, while whiskers indicate maximum and minimum values. **C** Viral transcript counts for VSV *gp5* during the course of VSV infection in transcripts per million (tpm). Mean values and SD of 3 technical replicates are depicted. **D** Titers of infectious viral particles determined from cell-free supernatants of VSV-infected WT and *Mavs*^*−/−*^ microglia (MOI 0.5). *N* = 2 independent experiments for each genotype, *n* = 2–3 technical replicates of each experiment. Mean with SD are depicted with individual values. **E** Spectral flow cytometric analysis of VSV-eGFP-infected WT and *Mavs*^*−/−*^ microglia cultures at 8 hpi (MOI 0.5). Histogram of live, single cells of one representative preparation per genotype is shown. **F** Quantification of VSV-eGFP-positive cells among live, single cells of VSV-eGFP-infected WT and *Mavs*^*−/−*^ microglia. *N* = 2 independent experiments for each genotype, *n* = 2–3 technical replicates of each experiment. Mean with SD are depicted with individual values. Statistical analyses were performed using multiple unpaired t test and asterisks indicate significant differences (**p < 0.01)

The dysregulation of GO terms corresponding to cytokine responses in *Mavs*^*−/−*^ microglia prompted us to probe for specific cytokine signatures influencing the phenotype. To this end, we selected all regulated cytokines and evaluated their expression levels. Comparative analysis revealed lower fold-induction of genes encoding for pro-inflammatory cytokines such as *Il6*, *Il12b*, *Il1b*, *Il19*, *Tnf*, *Il1a*, and *Il27* in *Mavs*^*−/−*^ microglia than in WT samples (Fig. 4A). This suggests that dysfunctional or delayed responses were induced in *Mavs*^*−/−*^ microglia upon VSV exposure when compared with WT microglia. Additionally, probing for the anti-inflammatory cytokines revealed higher induction of *Il10* in WT microglia than in *Mavs*^*−/−*^ microglia upon VSV infection. On the other hand, *Mavs*^*−/−*^ microglia showed more pronounced expression of *Tgfb2* than WT microglia. Moreover, genes involved in antigen presentation (e.g., *Fcgr1*, *Tap1*, and *Cd40*) gradually increased over the course of infection in WT microglia, whereas in *Mavs*^*−/−*^ microglia they remained unchanged (Fig. 4B, Fig. S2D). Considering the defective cytokine responses and impaired activation of VSV-exposed *Mavs*^*−/−*^ microglia, we next analyzed the expression of viral genes. To this end, fastq files of VSV-infected samples were mapped onto the VSV genome. Indeed, numbers of VSV mRNAs were considerably enhanced in *Mavs*^*−/−*^ microglia when compared with WT microglia during the course of VSV infection except for the 6 hpi timepoint (Fig. 4C). To further verify defects in viral restriction in *Mavs*^*−/−*^ microglia, we infected microglia with VSV in vitro and evaluated viral titers in the cell-free supernatant by plaque assay. Indeed, the supernatants of *Mavs*^*−/−*^ microglia contained higher viral loads than their WT counterparts (Fig. 4D). Infection with VSV-eGFP revealed that a significantly higher percentage of *Mavs*^*−/−*^ microglia supported virus gene expression than WT microglia (Fig. 4E-F). Collectively, these data indicated that while WT microglia were largely resistant to VSV infection, *Mavs*^*−/−*^ microglia showed a significantly enhanced susceptibility to virus infection. Thus, in microglia cultures MAVS is crucially involved in the restriction of VSV propagation.

### Upon intranasal VSV instillation of mice, MAVS signaling is essential for virus control in the CNS

**Fig. 5:**
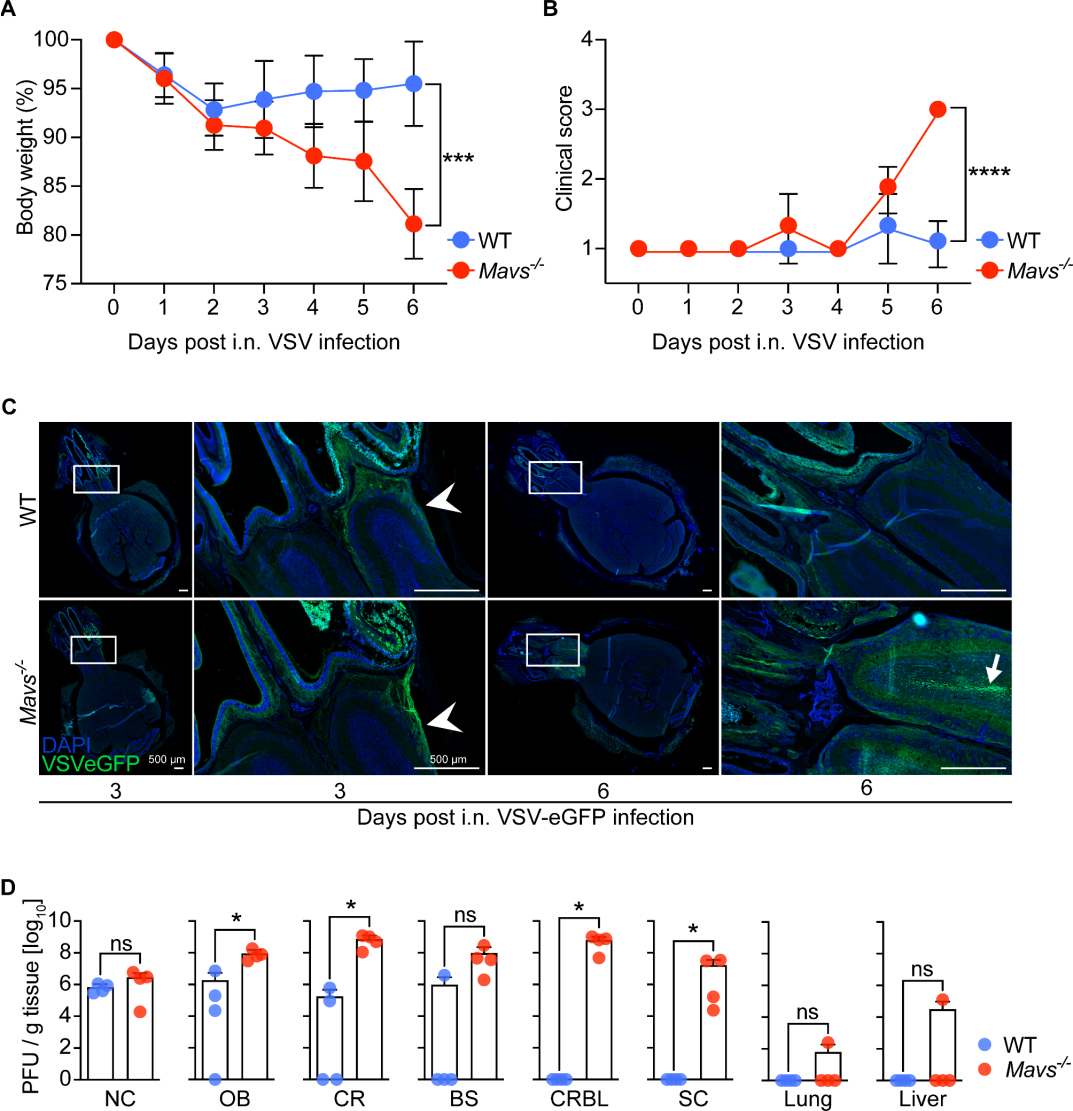
After intranasal VSV installation of mice, MAVS is essential for virus control and protection. **A**, **B** WT (C57BL/6) mice and *Mavs*-deficient mice (*Mavs*^*−/−*^) were instilled with 1000 PFU VSV intranasally and (**A**) body weight and (**B**) clinical score were monitored (*n* = 8–9). **C** Whole head sections of WT or *Mavs*^*−/−*^ mice i.n. infected with VSV-eGFP (1000 PFU) for 3 or 6 days. 16–20 μm thick sections were counterstained with DAPI (AxioScan, objective 10X, stitched image). Zoom shows the intersection of the nasal cavity (NC) to the olfactory bulb (OB). Arrows indicate invasion of VSV-eGFP in the glomerular layer (➤) and the granular cell layer (➞) of the OB (*n* = 3). **D** Titers of infectious viral particles determined from homogenized brain tissues from VSV-infected mice (WT and *Mavs*^*−/−*^, 1000 PFU) show higher infiltration of VSV in more distal regions than the OB in *Mavs*-deficient mice. NC, nasal cavity, OB, olfactory bulb; CR, cerebrum; CRBL, cerebellum; BS, brainstem; SC, spinal cord (*n* = 4). (**A**,** B**) Log-rank (Mantel Cox) test, (**D**) Unpaired t-test. *p < 0.05, ***p < 0.001, ****p < 0.0001, ns = not significant

Since pathway analysis of microglia cultures revealed a functional role of MAVS in mounting antiviral defense responses and responses to IFN-β (Fig. 3C-D) and in restricting virus replication in microglia cultures (Fig. 4C-F), we next sought to investigate the role of MAVS in controlling virus dissemination within the infected CNS under in vivo conditions. Upon i.n. VSV instillation, WT mice maintained stable body weight and showed low clinical scores (Fig. 5A-B). In contrast, VSV-infected *Mavs*-deficient mice lost weight, developed high clinical scores, and reached a critical status that required termination of the experiment by day 6 after infection (Fig. 5A-B). To address whether excessive virus propagation was responsible for the detrimental outcome of VSV infection in *Mavs*^*−/−*^ mice, we administered VSV-eGFP i.n. and monitored virus dissemination in the brain by fluorescence microscopy (Fig. 5C). Remarkably, in WT mice virus spread was restricted to the nasal cavity (NC) and to the glomerular layer of the olfactory bulb (OB) at 3 dpi, while at 6 dpi the virus was largely cleared from the entire OB. In contrast, in *Mavs*-deficient mice we observed slightly more eGFP signal in the OB at 3 dpi than in WT animals, while at 6 dpi we detected higher levels of viral neuroinvasion within the OB reaching the granular cell layer. Virus loads in different brain regions were further examined by plaque assays (Fig. 5D). 6 days following i.n. VSV infection, viral titers in the NC were similar in WT and *Mavs*^*−/−*^ mice, indicating a comparably effective virus control within the NC. Within the CNS, *Mavs*-deficient mice showed significantly enhanced viral loads in almost all brain regions. In the OB and the cerebrum (CR), WT mice exhibited significantly lower viral loads than *Mavs*-deficient mice, whereas in the brain stem (BS) only one out of four WT mice showed elevated virus titers, while all *Mavs*^*−/−*^ mice contained high viral loads (Fig. 5D). Interestingly, in the distal brain regions, i.e., the cerebellum (CRBL) and the spinal cord (SC), no infectious particles were detected in WT mice, whereas in *Mavs*^*−/−*^ mice high amounts of infectious virus particles were present (Fig. 5D). Virus spread throughout the CNS in *Mavs*^*−/−*^ mice indicates failed control of the virus infection in the NC and brain regions that are closer to the site of instillation. Infectious virus was undetectable in the lung and the liver of WT mice and could be detected only in one out of four *Mavs*^*−/−*^ mice, which was not significant. In summary, this data shows that MAVS signaling is crucial for viral restriction and containment of the infection in the NC and proximal regions of the CNS.

### Highly activated non-microglial myeloid cells infiltrate the OB of *Mavs*-deficient mice upon i.n. VSV infection

**Fig. 6:**
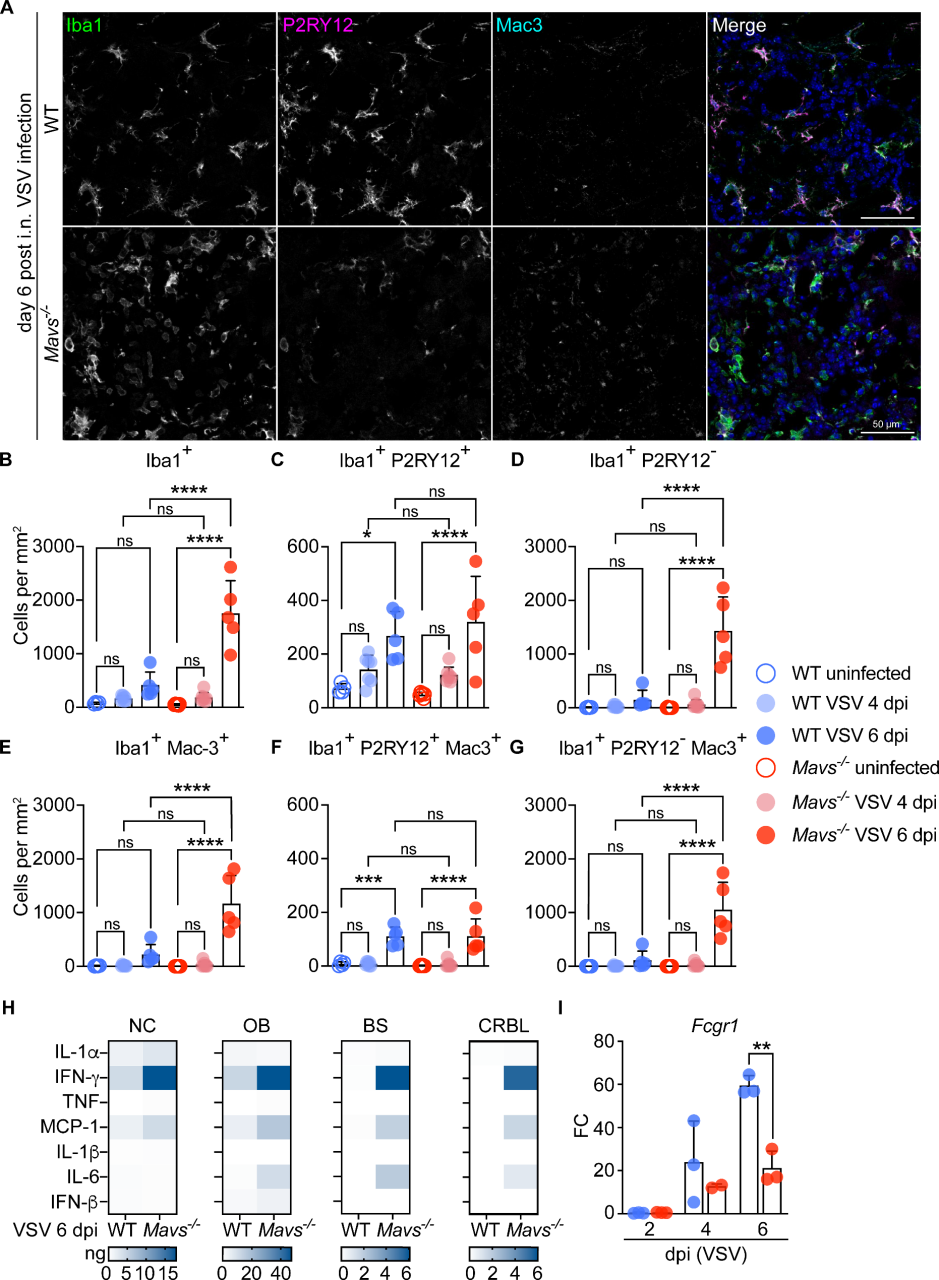
Exacerbated and ineffective inflammatory responses are induced in the brain of VSV-infected *Mavs*^*−/−*^ mice. WT or *Mavs*^*−/−*^ mice were i.n. infected with VSV (1000 PFU) for 4 or 6 days prior to organ collection. **A-G** 7 μm thick brain sections from VSV-infected WT or *Mavs*^*−/−*^ mice were immunolabeled for Iba1, P2RY12, and Mac-3, and counterstained with DAPI. Confocal immunofluorescence microscopy was performed. **A** Representative images of the OB show more pronounced increase of Iba1^+^ P2RY12^−^ Mac-3^+^ cells in *Mavs*^*−/−*^ mice 6 days after VSV infection (Olympus Fluoview 3000, objective 20x, stitched image). **B-D** Quantification of (**B**) Iba1^+^, (**C**) Iba1^+^ P2RY12^+^, and (**D**) Iba1^+^ P2RY12^−^ cells in the glomerular layer of the OB at 6 dpi. **E-G** Quantification of (**E**) Iba1^+^ Mac-3^+^, (**F**) Iba1^+^ P2RY12^+^ Mac-3^+^, and (**G**) Iba1^+^ P2RY12^−^ Mac-3^+^ cells per mm^2^ of the glomerular layer of the OB at 6 dpi (*N* = 1–2, *n* = 4–7, mean values and SD depicted). **H** Quantification of cytokines (ng cytokine / g tissue) in homogenates of the NC, OB, BS, and CRBL from VSV-infected WT and *Mavs*^*−/*−^ mice at day 6 post infection (*n* = 4, mean values). **I** Fold change (FC) of *Fcgr1* transcripts in the OB as determined by RT-qPCR at day 2, 4, and 6 days post i.n. VSV infection (1000 PFU) compared to uninfected controls (*N* = 1, *n* = 2–3). **(B-G)** Ordinary one-way ANOVA. **(I)** Unpaired t-test. **p < 0.01, ***p < 0.001, ****p < 0.0001, ns = not significant

We next analyzed myeloid cell responses in the OB of WT and *Mavs*^*−/−*^ mice. To differentiate between infiltrating myeloid cells and CNS-resident microglia, we used P2RY12 as a reliable and specific marker of microglia [35, 37–41]. While P2RY12 may be diminished under inflammatory conditions [38], its expression is still sufficient for differentiation of infiltrating myeloid cells and microglia, even under inflammatory conditions after virus infection [21, 42]. P2RY12 is selectively expressed in activated platelets [43] and in microglia in the brain [35, 44]. Importantly, macrophages and monocytes express low to undetectable levels of P2RY12 [45, 46]. To further define bone-marrow-derived infiltrating myeloid cells, we analyzed Mac-2 as a well-characterized marker that often is used in transcriptomic and protein analyses [47]. We first confirmed absence of Mac-2 in the vast majority of P2RY12^+^ cells at day 6 post i.n. VSV infection confirming the adequacy of P2RY12 labeling for discrimination of microglia and infiltrating myeloid cells (Fig. S3A). Secondly, we observed no significant decline in *P2ry12* expression levels in the OB bulk RNA in WT and *Mavs*^*−/−*^ mice during the course of infection, as determined at day 2, 4, and 6 post i.n. VSV infection, indicating overall rather stable *P2ry12* expression (Fig. S3B). OBs from uninfected WT and *Mavs*^*−/−*^ mice contained similar numbers of Iba1^+^ myeloid cells, which increased at day 6 post i.n. VSV infection, with a stronger increase in *Mavs*^*−/−*^ than in WT mice (Fig. 6A-B, Fig. S3C). We next aimed to determine whether this increase was mediated by the proliferation of resident microglia within the CNS or by enhanced infiltration with myeloid cells. In uninfected mice, myeloid cells were primarily microglia as indicated by their Iba1^+^ and P2RY12^+^ status (Fig. 6C and Fig. S3C). Their abundance increased similarly in WT and in *Mavs*^*−/−*^ animals at day 6 post infection. While in the OB of uninfected mice Iba1^+^ P2RY12^−^ cells were absent, these cells were detected in the OB of WT and *Mavs*^*−/−*^ mice on 6 dpi, whereas overall numbers of these cells were significantly higher in *Mavs*^*−/−*^ mice (Fig. 6A and D). All significant changes occurred at day 6 post VSV infection, while at 4 dpi numbers were not significantly different in infected mice and controls, further highlighting the peak of myeloid cell infiltration at 6 dpi in the glomerular layer of the OB. Thus, while Iba1^+^ P2RY12^+^ microglia numbers increased similarly in WT and *Mavs*-deficient mice by day 6 post VSV infection, infiltration with Iba1^+^ P2RY12^−^ myeloid cells into the infected OB was exacerbated in the absence of *Mavs*.

To investigate the activation status of myeloid cells in the OB, we assessed Mac-3 expression. Under homeostatic conditions, the activation marker Mac-3 was not detectable in the OB, neither of WT nor *Mavs*^*−/−*^ mice (Fig. S3B and Fig. 6E). In contrast, at 6 days post VSV infection, Mac-3 expression was induced on Iba1^+^ myeloid cells in the OB of WT and *Mavs*^*−/−*^ mice, while the Mac-3 induction detected in *Mavs*^*−/−*^ mice was stronger than in WT controls (Fig. 6A and E). Interestingly, while no statistical difference of Iba1^+^ P2RY12^+^ cells expressing Mac-3 in the OB of WT and *Mavs*-deficient mice was detected upon VSV infection, Iba1^+^ P2RY12^−^ myeloid cells showing Mac-3 expression were strongly enhanced in *Mavs*-deficient mice (Fig. 6A and F-G). Thus, absence of MAVS signaling leads to increased infiltration of the OB with Iba1^+^ P2RY12^−^ non-microglia myeloid cells that show an enhanced inflammatory phenotype.

Following up on the defective cytokine responses in *Mavs*-deficient microglia cultures, we profiled pro-inflammatory cytokines in the NC as well as in several CNS regions on day 6 after infection. In the NC, several cytokines including IL-1α, IFN-γ, and MCP-1 were detected in WT and *Mavs*^*−/−*^ mice, which were higher in *Mavs*-deficient animals (Fig. 6H). Similarly, in the OB of *Mavs*-deficient mice IFN-γ, MCP-1, IL-6, and IFN-β were detected at higher levels than in WT mice. In the BS and the CRBL, none of the analyzed cytokines was detected in WT mice, whereas *Mavs*-deficient mice had high levels of IFN-γ, MCP-1, and IL-6 in these brain regions. Inspired by the in vitro transcriptomic data, we next profiled *Fcgr1* transcript levels in the OB of WT and *Mavs*-deficient animals upon i.n. VSV infection. In line with the in vitro data, *Fcgr1* levels increased between 4 and 6 dpi in WT animals, while in *Mavs*-deficient mice this increase was less pronounced (Fig. 6I). Thus, the CNS of VSV-infected *Mavs*-deficient mice is excessively inflamed as indicated by the presence of highly activated infiltrating non-microglial myeloid cells and profound levels of pro-inflammatory cytokines, while expression of important mediators of the immune response such as *Fcgr1* are not potently induced.

## Discussion

During viral encephalitis, CNS-resident cells are key modulators of immune responses, however, to which extent and by triggering of which pathways different cell types in the brain contribute to virus control is still poorly understood. Here, we studied microglial responses to neurotropic VSV infection in in vitro as well as in vivo settings. We validated murine primary cultured microglia as a suitable model to investigate antiviral responses of microglia without the influence of other cell types in the direct environment. Microglia cultures responded to VSV exposure in a time-dependent manner. Initially, exclusively pro-inflammatory pathways were induced, followed by the induction of both activating and regulating pathways, demonstrating a highly regulated response to VSV infection. In contrast, *Mavs*-deficient microglia showed dysregulated inflammatory responses and defective cytokine induction, which resulted in enhanced virus replication as indicated by increased numbers of viral transcripts, increased virus titers in the supernatants of infected cells as well as higher percentages of microglia that supported viral gene expression. Indeed, i.n. VSV infection of *Mavs*-deficient mice resulted in virus spread throughout the whole CNS. Although microglia frequencies and their activation in the OB were similar in WT and *Mavs*^−/−^ mice, a highly activated population of non-microglial myeloid cells infiltrated the brain of *Mavs*-deficient mice. Consequently, increased cytokine levels correlated with virus dissemination and myeloid cell infiltration in *Mavs*-deficient animals.

Microglia cultures showed expression of several characteristic myeloid and microglia markers, while marker genes for astrocytes, oligodendrocytes, and neurons were absent. However, several key microglia markers typically found in the CNS of whole organisms including *Tmem119*,* P2ry12*, and *Csf1r* were only expressed at low levels or not at all in microglia cultures. Detection of abundant *Csf1r* gene expression, but only very low CSF-1R protein expression, is common for immature developmental stages of macrophages (common myeloid progenitors). *Csf1r* expression translates into high surface CSF-1R protein expression in the final differentiation stages of macrophages [48]. Microglia isolated from neonates are still in the maturating process and have not yet acquired the final differentiated state. The decreased expression of microglia-specific markers in microglia under inflammatory conditions [21] and microglia extracted from the CNS has been reported previously [49], while microglia derived from newborn mice still showed the highest expression of these markers when compared with other in vitro microglia models, such as microglia cell lines [35]. Despite the lack of several microglia markers, primary murine microglia are capable of exerting important microglia functions, such as phagocytosis [50], production of cytokines, chemokines, and growth factors [51], as well as interaction with T cells and subsequent upregulation of activation markers [29]. Hence, the murine primary microglia cultures used here are a robust model to assess changes after VSV exposure without the influence of other cell types.

Microglia are key effectors in viral encephalitis that critically determine disease outcome [52]. Microglia express a variety of PRRs including Toll-like receptors (TLRs) [53] and RLRs [36] and are among the first cells that respond to CNS insults. Microglia are constantly vigilant and surveil their local environment under homeostatic conditions [15]. While many studies analyzed microglial responses under conditions of sterile inflammation, here we addressed kinetics of microglia responses to VSV infection. Pathways that were induced within a short time after virus exposure included response to virus infection and IFN-β, which was followed by both activating and regulatory pathways pointing towards a balanced immune response. *Ptpn2* and *Nr1d1* were found among the upregulated immunomodulatory genes after VSV infection. *Ptpn2* has been shown to be responsible for an anti-inflammatory phenotype in microglia [54] and to inhibit JAK-STAT signaling in various different cell types including neurons [55]. Triggering of REV-ERBα, the protein corresponding to the *Nr1d1* gene product, leads to NF-κB inhibition followed by a reduced inflammatory phenotype in microglia cultures [56]. This tight regulation of the immune response has been demonstrated by several studies. On the one hand, in several infection models, e.g., with VSV and WNV, microglia presence was indispensable for protection [21, 24]. On the other hand, microglial inflammatory signaling was responsible for neuronal death during JEV, Zika virus (ZIKV), and WNV infection [57, 58]. The in vitro microglia infection model presented here is thus highly sensitive and multifaceted, further highlighting the key relevance of the regulation of an otherwise detrimental response by microglia.

RLR-MAVS signaling is fundamental for the control of RNA-encoded viruses [9]. The relevance of this sensing pathway has been demonstrated in human cell lines [9], mouse models [27, 59, 60], and murine cell cultures [61, 62] as well as in human individuals with primary immunodeficiencies [63]. Here, we confirm the importance of RLR-MAVS signaling in microglia to mount IFN and cytokine responses upon RNA virus infection and to induce a variety of immune pathways that restrict virus replication within microglia including innate immune activation, cytokine production and response to cytokines, response to virus, and T-cell activation. RIG-I has previously been reported to contribute to microglial cytokine responses to VSV [36]. Triggering of pro-inflammatory cytokine responses is dependent on successful infection of microglia with viable VSV particles [64]. Indeed, in our system we observed that microglia cultures were productively infected and while viral transcripts within microglia increased, transcriptional programs of antiviral responses were initiated. Conversely, in *Mavs*-deficient microglia, VSV replication was enhanced while antiviral responses were delayed and reduced. Several IFNs and ISGs exhibit antiviral effects, as demonstrated for IFN-α against neurovirulent Venezuelan equine encephalitis virus infection [65], Ifi27l2a in neurons and macrophages against WNV infection [66], IFIT1 against VSV and Rift Valley fever virus (RVFV) infection [67], ISG54 against neurovirulent VSV [68] and many others [69]. Several viruses developed immune evasion strategies by inhibiting MAVS signaling by downregulation [70], cleavage [71, 72] or autophagic degradation [73] of MAVS, or impeding MAVS interaction with upstream receptors [74]. Suppressed MAVS signaling results in enhanced virus replication [73]. Hence, MAVS signaling is of central importance for antiviral defenses, and defective antiviral signatures in *Mavs*-deficient microglia might contribute to failed virus control.

Here, we found enhanced CNS infiltration of non-microglia myeloid cells in *Mavs*-deficient animals upon VSV infection. Myeloid cell responses in the CNS can be investigated by several techniques including flow cytometry and fluorescence microscopy. A major advantage of flow cytometry is the multi-parameter analysis of cells within the infected CNS with the possibility to separate cell populations according to the expression of sets of surface markers [75]. Additionally, expression of activation markers such as co-stimulatory molecules can be addressed within distinct subsets [75]. However, flow cytometry has its limitations as tissue disintegration is needed to prepare single-cell suspensions from brain tissue, which typically involves enzymatic digestion. These experimental procedures can cause cellular stress and skewing of the expression of activation markers [76]. Moreover, several enzymes used for tissue digestion, including collagenase, can cleave surface markers, which potentially affects readouts [77]. Microscopic inspection of tissue allows the analysis of the anatomical localization of cells, the expression of cellular markers, and the cell morphology without the need to lyse tissue. Therefore, we decided to study the integer CNS tissue in this study and to analyze cellular marker expression by fluorescence microscopy. The identity of microglia and non-microglia myeloid cells was determined by Iba1 and P2RY12 co-expression of microglia and Iba1 expression alone of non-microglia myeloid cells. We further confirmed the accurate assignment of cell types by P2RY12 / Mac-2 co-labeling, which faithfully discriminates microglia and monocytes / macrophages. Indeed, the vast majority of P2RY12^+^ cells were negative for Mac-2. A small proportion (< 5%) of cells were positive for both P2RY12 and Mac-2 which might account for a recently discovered specialized subset of microglia with a progenitor-like phenotype which remains to be further analysed in detail [78]. Upon VSV infection, microglia and non-microglia myeloid cells mounted Mac-3 expression, indicating a responsive cell status as illustrated in several models of neuroinflammation such as aging [79] or during infection [21]. Upon intraperitoneal Langat virus (LGTV) infection, a similar increased infiltration of myeloid cells into the CNS was observed in *Mavs*-deficient mice, which correlated with increased cytokine levels and virus titers in the CNS [80]. In contrast, upon i.n. RVFV infection, moderately reduced infiltration of myeloid cells was noticed in *Mavs*-deficient mice [27]. Upon intracranial LGTV infection, recruitment of peripheral monocytes and macrophages to the CNS was almost entirely abolished in *Ifnar*-deficient mice [81]. Thus, different compensatory mechanisms might be present in the VSV model when compared with the RVFV and LGTV models. We have previously shown that after i.n. VSV instillation recruitment of peripheral immune cells was dependent on MyD88 signaling of neurons [82], which should be undisturbed in *Mavs*-deficient mice. In fact, enhanced virus replication of neurons in *Mavs*-deficient mice might enhance triggering of other PRRs. Especially TLR engagement and subsequent MyD88 signaling in neurons might increase neuronal chemokine responses, which in turn would enhance recruitment of highly activated infiltrating monocytes and/or macrophages as a tentative compensatory mechanism. Despite compensatory mechanisms being in place, microglia are indispensable for the control of CNS virus infection. In mouse models of microglia depletion, mice are drastically more vulnerable to viral encephalitis despite infiltration of myeloid cells [23]. In the VSV encephalitis model, the cross-presenting capacity of microglia to antigen-specific CD8^+^ cytotoxic T cells was necessary for protection [83]. In the RVFV infection model, *Mavs*^*−/−*^ mice contained higher numbers of T cells and NK cells in the infected CNS, however, gene counts for *Gzmb* and *Ifng* were slightly lower than in WT mice, suggesting inefficient lymphocyte activation [27]. Infiltrating myeloid cell populations contain a different set of surface markers, which are involved in antigen presentation, including higher expression of CD80 [84], CD86 [21, 84], and MHC-II [84] and have been described to be involved in eliciting CD4^+^ T-cell responses [84]. Here, we observe a highly inflammatory response without viral clearance. MAVS might therefore be involved in the full microglia activation to specifically restimulate cytotoxic T cells and thus retain the appropriate T-cell stimulation level that is needed for viral clearance. Defective induction of *Fcgr1* expression upon VSV infection of *Mavs*^−/− ^in vitro microglia as well as in the OB of *Mavs*^−/−^ mice might have consequences on the efficient antigen uptake and subsequent antigen presentation to T cells. In a mouse lung infection model with pneumonia virus, *Fcgr1* expression in inflammatory type 2 dendritic cells was linked with superior CD4^+^ T-cell polarization as well as antigen presentation to CD8^+^ T cells [85]. Within the CNS, this FCGR1-triggered enhanced T-cell crosstalk might be conferred by microglia, through increased microglial *Fcgr1* expression in a MAVS-dependent manner, which leads to efficient and protective responses [86].

Here, we report productive VSV infection in WT microglia cultures, which is dramatically enhanced in absence of *Mavs*. Since under in vivo conditions microglia are largely resistant to RNA virus infection [61], it is possible that in *Mavs*-deficient mice only moderately enhanced virus infection of microglia is detected. Interestingly, in the mouse model of i.n. RVFV infection, microglia were not infected in fully immunocompetent mice, while productive virus infection could be detected in microglia of *Mavs*^*−/−*^ mice [27]. Moreover, upon intracranial LGTV infection, it was shown that *Ifnar* deficiency shifts the viral tropism from neurons to microglia with a complete loss of myeloid cell infiltration within the infected CNS [81]. Infected *Ifnar*-deficient microglia are still able to sense the virus and to activate downstream IFN-independent signaling cascades that are essential for tissue immunopathology. Under fully immunocompetent conditions, IFNAR stimulation induces upregulation of the viral RNA sensor RIG-I. In absence of IFNAR, RIG-I upregulation is impaired and might in turn contribute to further reduced virus sensing. *Ifnar* deficiency affects several different immune sensing pathways. In complete *Ifnar*-deficient mice, MyD88-triggered IFN signaling in neurons is additionally impaired, which could be the reason for the defective immune cell infiltration in such mice. On the contrary, in the i.n. VSV infection model presented here, *Mavs*^−/−^ mice exhibited enhanced infiltration with peripheral myeloid cells. We therefore hypothesize that MAVS signaling in microglia is crucial for the orchestration of protective antiviral responses, presumably through effective T-cell restimulation and the regulation of myeloid cell infiltration of the infected CNS, possibly by an interplay with other CNS-resident cells such as astrocytes and neurons. Under in vivo conditions, complete *Mavs* deficiency can have several indirect effects that are contributed by other cells than microglia. Astrocytes require MAVS to mount protective IFN-β responses upon TBEV infection [61], while *Mavs*-deficient monocytes failed to control WNV neuroinfection [86], and CD8^+^ T cells lacking MDA5 were unable to clear WNV infection in the CNS [87]. The precise mechanism of how *Mavs* deficiency confers microglia malfunction under in vivo conditions remains to be further clarified and future experiments using microglia-specific *Mavs* knockout might further illuminate specific mechanisms. However, the integrated analysis of data derived from pure in vitro microglia cultures along with in vivo experiments point towards a crucial role of MAVS in microglia for the induction of protective antiviral responses during RNA virus infection of the brain.

## Conclusion

Our data indicate a crucial role of the RLR-MAVS signaling axis in the CNS for the control of neurotropic virus infection. RLR-MAVS signaling unleashes full microglia activation, which is needed to restrict virus replication in an in vitro setting. In vivo, RLR-MAVS signaling is required for efficient viral clearance from the infected brain and simultaneously limits excessive inflammatory responses within the CNS. In absence of *Mavs*, failed virus control correlates with excessive inflammation that is mediated by infiltrating myeloid cells, and that contributes to the detrimental disease outcome.

## Electronic supplementary material

Below is the link to the electronic supplementary material.

**Figure S1** Gating strategy for in vitro microglia flow cytometric analysis. **A**, **B** Immunofluorescent confocal microscopy of isolated (**A**) in vitro microglia and (**B**) astrocytes immunolabeled with Iba1 (red) and GFAP (green) and counterstained with DAPI (cyan). Objective 20x. **C** Gating strategy of in vitro microglia for flow cytometric analyses by hierarchically gating cells, singlets, and live cells

**Figure S2** Microglia responses to in vitro VSV infection are dysregulated in the absence of *Mavs*. **A**,** B***Mavs*^*−/−*^ microglia were isolated, plated, infected with VSV at an MOI of 0.5 or mock-treated (Ø). Cells were harvested and total RNA was isolated to perform bulk RNA-seq analysis. **A** Heatmap of k-means clustering of DEGs, (log_2_ fold-change > │2│, padj < 0.05) of *Mavs*^*−/−*^ microglia. Each column represents transcripts from a technical replicate. **B** Pathway analysis of 4 clusters visualized in the heatmap. Cluster I and II comprise genes that are upregulated after VSV infection; cluster III and IV comprise genes that are downregulated after VSV infection. *n* = 3 technical replicates. **C** Numbers of differentially regulated genes (DEGs) in VSV-infected WT and *Mavs*^*−/− *^in vitro microglia compared to the respective mock-treated control (Ø). Positive values indicate upregulated genes, while negative values indicate downregulated genes. **D** Normalized gene counts of *Tap1* and *Cd40* of VSV-infected or mock-treated (Ø) WT and *Mavs*-deficient microglia. Each group contains triplicates. Each box represents interquartile range, while whiskers indicate maximum and minimum values

**Figure S3** Expression of microglia markers upon i.n. VSV infection of mice. **A-C** WT or *Mavs*^*−/−*^ mice were i.n. infected with VSV (1000 PFU) for 6 days prior to organ collection. **A** 16–20 μm thick brain sections from VSV-infected mice were immunolabeled for Iba1, P2RY12, and Mac-2, and counterstained with DAPI. Confocal immunofluorescence microscopy was performed (Olympus Fluoview 3000, objective 20x, stitched image). Representative images of the OB show low degree of co-localization of P2RY12 and Mac-2 within cells. *N* = 2, *n* = 5. **B** Fold change (FC) of *P2ry12* transcripts in the OB bulk RNA determined by RT-qPCR at day 2, 4, and 6 days post i.n. VSV infection (1000 PFU) compared to uninfected controls. *n* = 2–3. **C** Representative images of the OB show more pronounced increase of Iba1^+^ P2RY12^−^ Mac-3^+^ cells in *Mavs*^*−/−*^ mice 6 days after VSV infection (objective 20x, stitched image). 7 μm thick brain sections from VSV-infected WT or *Mavs*^*−/−*^ mice were immunolabeled for Iba1, P2RY12 and Mac-3, and counterstained with DAPI and confocal immunofluorescence microscopy was performed (VSV, 1000 PFU, i.n., objective 20x). **(B)** Ordinary one-way ANOVA, ns = not significant

## Data Availability

All RNA-seq data is available under the Gene Expression Omnibus (GEO) accession numbers GSE274915 and GSE271293. The authors confirm that all data underlying the findings are fully available without restriction upon request to the lead contact.

## Abbreviations

Adar: Adenosine deaminase
Aldh1l1: Aldehyde dehydrogenase 1 family member L1
AP-1: Activator protein 1
BP: Biological processes
BS: Brain stem
C: Celsius
CCL CC: Motif chemokine ligand
CCR CC: Motif chemokine receptor
CD: Cluster of differentiation
CNS: Central nervous system
CR: Cerebrum
CRBL: Cerebellum
CSF1R: Colony stimulating factor 1 receptor
CX3CR1 C-X3-C: Motif chemokine receptor 1
DAPI: 4′,6-diamino-2-phenylindole
DNA: Deoxyribonucleic acid
Dusp7: Dual specificity phosphatase 7
eGFP: Enhanced green fluorescent protein
Fcgr: Fc-gamma receptor
FCS: Fetal calf serum
FDR: False Discovery Rate
Fut7: Fucosyltransferase 7
GFAP: Glial fibrillary acidic protein
GLAST: Glutamate–aspartate transporter
GO: Gene ontology
GSEA: Gene set enrichment analysis
H: Hour(s)
Hpi: Hours post infection
Iba1: Ionized calcium-binding adapter molecule
Icosl: Inducible T Cell Costimulator Ligand
Ifi27l2a: Interferon alpha-inducible protein 27 like 2 A
IFN: Interferon
IFN-I: Type I interferon(s)
IFNAR: Interferon α/β receptor
IKKɛ: IκB kinase
IL: Interleukin
i.n.: Intranasal
IRF: Interferon regulatory transcription factor
ISG: Interferon stimulated gene
JAK: Janus kinase
JEV: Japanese encephalitis virus
LGP2: Laboratory of genetics and physiology 2
LGTV: Langat virus
M: Milli
MAVS: Mitochondrial antiviral-signaling protein
Map2: Microtubule-associated protein 2
Mbp: Myelin binding protein
Mcm4: Minichromosome maintenance complex component 4
MCP-1: Monocyte chemoattractant protein 1
MDA5: Melanoma differentiation associated factor 5
MHC: Major histocompatibility complex
Min: Minute
mRNA: Messenger RNA
mL: Milliliter
Mmp11: Metalloproteinase 11
MOI: Multiplicity of infection
MyD88: Myeloid differentiation primary response 88
NC: Nasal cavity
Nes: Nestin
Ng: Nanogram
NF-κB: Nuclear factor ‘kappa-light-chain-enhancer’ of activated B-cells
Nr1d1: Nuclear receptor subfamily 1 group D member 1
n.s.: Not statistically significant
Oas1a: 2’-5’-oligoadenylate synthetase 1
OB: Olfactory bulb
Ocln: Occludin
P_ Day: Post-natal development (mouse)
P2RY12: Purinergic receptor P2Y12
PAMP: Pathogen associated molecular pattern
PBS: Phosphate-buffered saline
PC: Principal component
PCA: PC analysis
Pdgfra: Platelet-derived growth factor receptor alpha
PFA: Paraformaldehyde
PFU: Plaque forming unit
Plp1: Proteolipid protein 1
Prc1: Protein regulator of cytokinesis 1
PRR: Pattern recognition receptor
Ptpn2: Protein tyrosine phosphatase non-receptor type 2
RVFV: Rift Valley fever virus
RIG-I: Retinoic acid inducible gene I
RLR: RIG-I-like receptor
RNA: Ribonucleic acid
RNA-seq: RNA sequencing
Rpm: Revolutions per minute
RT: Room temperature
Sall1: Spalt Like Transcription Factor 1
Setd1a: SET domain containing 1 A, histone lysine methyltransferase
SD: Standard deviation
SiglecH: Sialic acid binding Ig-like lectin H
Ss: single stranded
STAT: Signal transducer and activator of transcription
Syp: Synaptophysin
Tap1: Transporter associated with antigen processing 1
TBK1: TANK binding kinase
Th: T helper
TLR: Toll like receptor
TMEM: Transmembrane protein
TNF: Tumor necrosis factor
TRAM: TRIF-related adaptor molecule
TRIF: TIR-domain-containing adapter-inducing interferon-β; Tpm: Transcripts per million
VSV: Vesicular stomatitis virus
WNV: West Nile virus
WT: Wild type (C57BL/6)
Zbp1: Z-DNA binding protein 1
ZIKV: Zika virus
