# Supplementary figures and images for: MAVS signaling shapes microglia responses to neurotropic virus infection
Source: J Neuroinflammation. 2024 Oct 18;21:264. doi: 10.1186/s12974-024-03258-6 (PMC11490141; doi:10.1186/s12974-024-03258-6)

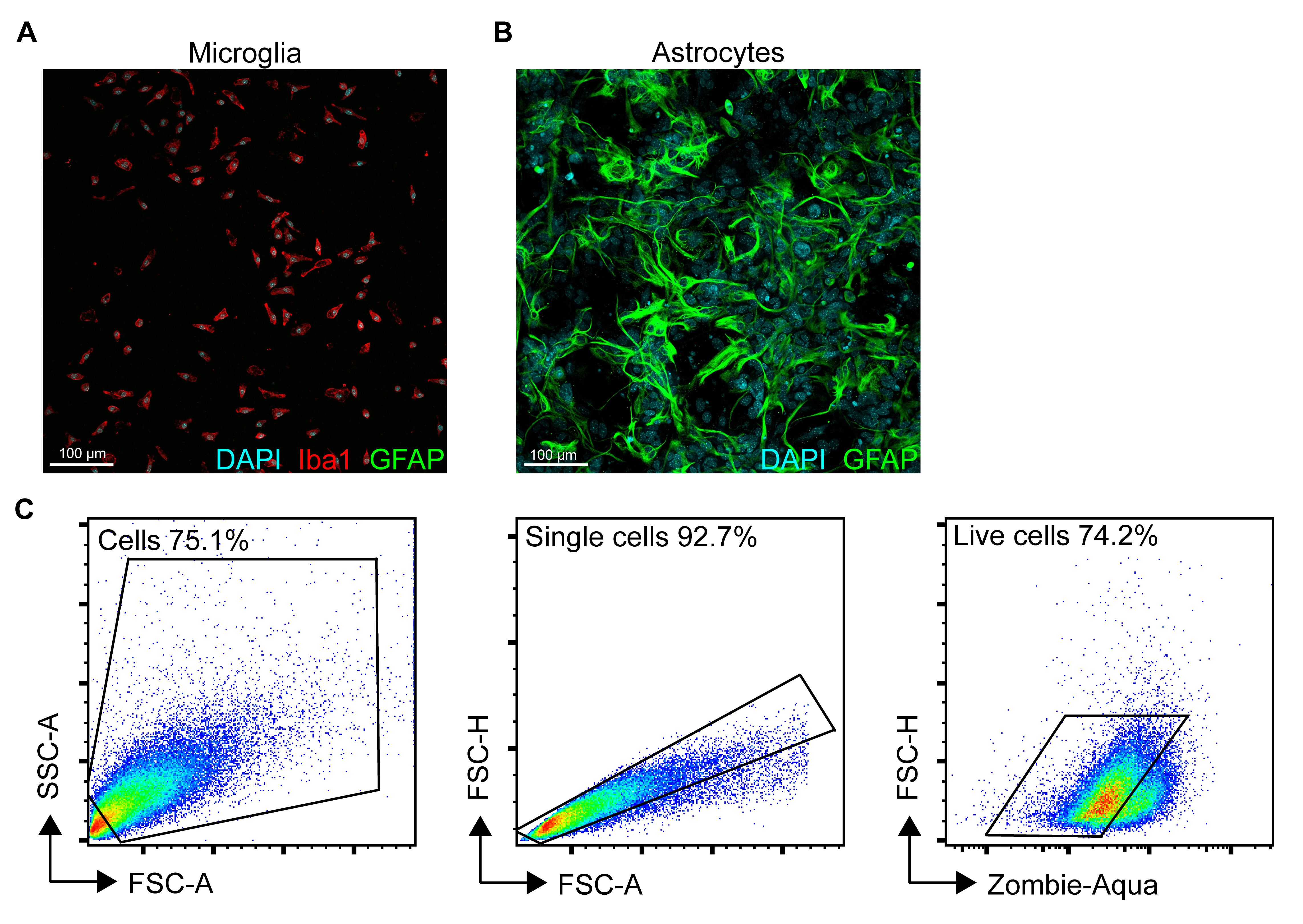

Supplement: Supplementary file 1 — Figure S1 Gating strategy for in vitro microglia flow cytometric analysis. A, B Immunofluorescent confocal microscopy of isolated (A) in vitro microglia and (B) astrocytes immunolabeled with Iba1 (red) and GFAP (green) and counterstained with DAPI (cyan). Objective 20x. C Gating strategy of in vitro microglia for flow cytometric analyses by hierarchically gating cells, singlets, and live cells [file 12974_2024_3258_MOESM1_ESM.tif]
